# The Reliability of the Volitional Questionnaire in Chinese (VQ-C) for Evaluating Motivation in Individuals with Schizophrenia

**DOI:** 10.1192/j.eurpsy.2025.2245

**Published:** 2025-08-26

**Authors:** W. Peng

**Affiliations:** Taipei City Hospital, Taipei, Taiwan, Province of China

## Abstract

**Introduction:**

Schizophrenia is a common diagnosis among individuals with mental health disorders, characterized by chronic mental illness that affects perception, volition, and emotions. Numerous studies have demonstrated that schizophrenia significantly reduces global functioning and impairs function. Motivation deficit, a core symptom in individuals with schizophrenia, directly or indirectly impacts their functioning and disease outcomes. Currently, there are limited assessment tools available for measuring motivation, especially in Chinese. The fourth edition of the Volitional Questionnaire in Chinese (VQ-C), developed based on the Model of Human Occupation (MOHO), assesses motivation levels in individuals. It is scored through activity observations of clients, providing a simple and clinically feasible evaluation method. The English version of the VQ has proven to be a reliable and valid tool for individuals with mental illnesses. However, the psychometric properties of the VQ-C have not been extensively discussed in the literature.

**Objectives:**

This study aims to test the reliability of the VQ-C in patients with schizophrenia.

**Methods:**

We recruited 45 patients with chronic schizophrenia. The VQ-C assesses clients’ motivation by observing their participation in various activities within their environment. The VQ-C consists of fourteen items, each rated on a 4-point scale, with higher scores indicating higher levels of motivation. Two raters independently observed participants’ engagement in two different types of activities (static and dynamic) over one week and scored their motivation levels based on their performance. Two testers simultaneously observed participants’ performance and provided scores.

In the data analysis, we used descriptive statistics to analyze demographic data of the participants, such as age, gender, and length of illness. Internal consistency was assessed using Cronbach’s alpha.

**Results:**

We recruited 45 participants with schizophrenia for the study, with 43 completing the test. The mean age was 43.02 years, and the mean duration of illness was 21.93 years. Each participant was assessed in two different activity contexts, allowing for independent analysis of scores in the sports and art contexts. The analysis of scores in the sports context revealed a Cronbach’s alpha of 0.95, indicating high reliability. Similarly, the assessment scores in the art context showed a Cronbach’s alpha value of 0.96.

**Conclusions:**

This study investigated the internal reliability of the VQ-C in patients with schizophrenia, demonstrating strong reliability. Based on these results, it is suggested that the VQ-C can be used to assess the motivational state of patients with schizophrenia, facilitating the design and implementation of treatment interventions.

**Disclosure of Interest:**

None Declared

